# Right ventricular perforation, pneumothorax, and a pneumatocele by a pacemaker lead: a case report

**DOI:** 10.1186/s40981-021-00470-8

**Published:** 2021-09-09

**Authors:** Shihoko Iwata, Ayana Hirose, Ikue Furui, Takako Matsumoto, Makoto Ozaki, Yasuko Nagasaka

**Affiliations:** 1 Department of Anesthesiology, Tokyo Women’s Medical University Hospital, 8-1, Kawada-cho, Shinjuku-ku, Tokyo, 162-8666 Japan; 2 Department of Thoracic Surgery, Tokyo Women’s Medical University Medical Center East, 2-1-10, Nishiogu, Arakawa-ku, Tokyo, 116-8567 Japan

**Keywords:** Pacemaker lead, Cardiac perforation, Pneumothorax, Pneumatocele, Lung resection

## Abstract

**Background:**

Perforation of the right ventricle by a pacemaker lead is a rare and potentially life-threatening complication. We present a patient who developed right ventricular perforation, pneumothorax, and a cyst and underwent partial lung resection.

**Case presentation:**

A 94-year-old woman was diagnosed with sick sinus syndrome and underwent a dual-chamber permanent pacemaker implantation. The next day, pacing failed and chest radiography showed that the right ventricular lead was outside the cardiac silhouette. Computed tomography revealed that the lead had perforated the right ventricular apex, causing a left-sided pneumothorax and a cystic lesion at the site of pulmonary injury by the pacemaker lead. The patient underwent lung resection and a right ventricular lead extraction. Pathological analysis revealed the cystic lesion to be an acute pneumatocele.

**Conclusions:**

Pneumothorax and pneumatocele associated with right ventricular pacemaker lead perforation is extremely rare. In our case, a radical surgical intervention provided an excellent outcome.

## Background

Acute pacemaker lead perforation of the right ventricle is rare but accompanies potentially life-threatening complications such as cardiac tamponade, pneumothorax, hemothorax, or death [1]. When the rapid progression of pericardial effusion or neighboring organ injury result in hemodynamic instability, surgical management may be the best treatment option [2]. We present a patient who developed acute right ventricular (RV) perforation, pneumothorax, and a pneumatocele because of a RV pacemaker lead and underwent the lead removal after thoracotomy and partial lung resection two days after the pacemaker implantation.

## Case presentation

**Fig. 1:**
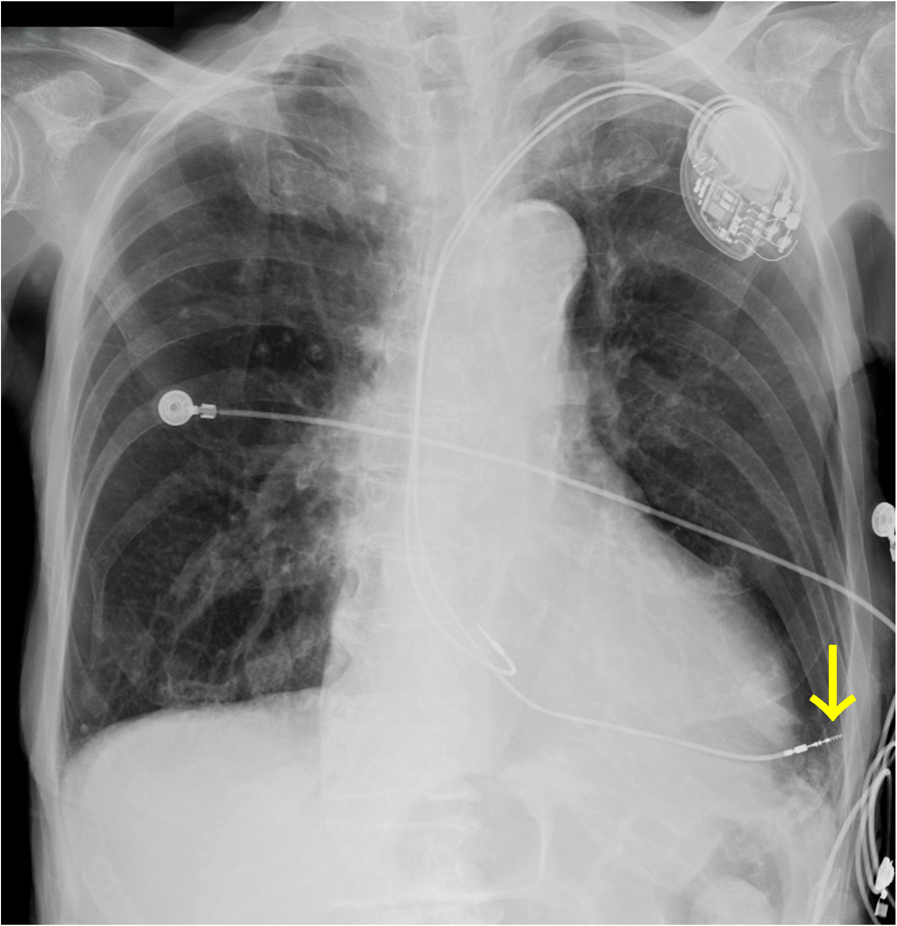
A preoperative chest radiograph. Chest radiograph showing the pacemaker lead perforating the right ventricle (yellow arrow)

**Fig. 2:**
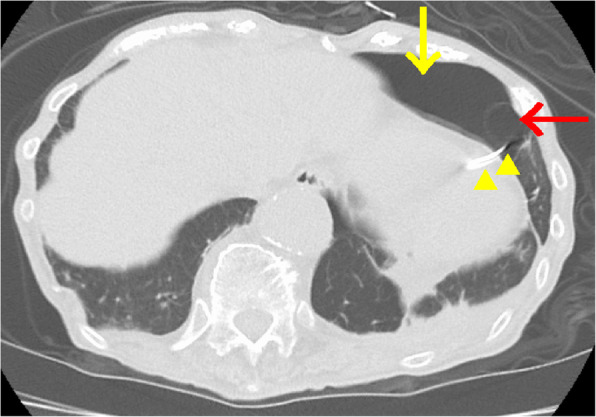
A preoperative chest computed tomography image. Thoracic computed tomography showing a left-sided pneumothorax (yellow arrow) and the pacemaker lead (yellow arrowheads) perforating the right ventricle and lung tissue neighboring a pulmonary cyst (red arrow)

A 94-year-old woman with a history of hypertension, congestive heart failure, and an abdominal aortic aneurysm (55 mm × 63 mm) presented with recurrent syncope episodes and ventricular pauses up to 8.1 s. Sick sinus syndrome was diagnosed, and she underwent a dual-chamber permanent pacemaker (Medtronic Japan Co., LTD., Tokyo, Japan) implantation via the left axillary vein at an outside hospital. The next day, fusion beats due to oversensing were seen on the electrocardiogram and capture threshold of the pacemaker was raised. Chest radiography was notable for the RV lead outside the cardiac silhouette (Fig. 1). Computed tomography (CT) revealed that the lead had perforated the RV apex, causing a left-sided pneumothorax (Fig. 2). The patient was transferred to our hospital for possible surgical intervention. The CT images demonstrated a cystic lesion (25 mm × 18 mm) of the left lung at the site of pulmonary injury by the pacemaker lead and associated pneumothorax (Fig. 2). The patient did not report any dyspnea and was hemodynamically stable; however, her SpO_2_ levels gradually decreased to 90% on room air and oxygen supplementation was started. A multidisciplinary team decided that immediate surgical intervention was necessary.

The patient was transferred to the operating room. Standard anesthesia monitoring was initiated, and arterial and central venous catheters were inserted. After induction of anesthesia, a left-sided double-lumen endotracheal tube (Broncho-Cath®, Mallinckrodt Medical, Inc., St Louis, MO, USA) was used for intubation and maintenance of anesthesia. One-lung ventilation was immediately started to prevent tension pneumothorax. Transesophageal echocardiography (TEE; Philips iE33 Ultrasound System, Philips Healthcare, Bothell, WA, USA) revealed two pacemaker leads; however, the location of the perforated myocardium was not identified. Mild aortic regurgitation, mild tricuspid regurgitation, mild reduction of left ventricular systolic motion, and a small pericardial effusion were noted. The pacemaker (DDD) was reprogrammed to asynchronous mode (AOO) at a heart rate of 80 for the usage of unipolar electrocautery.

**Fig. 3:**
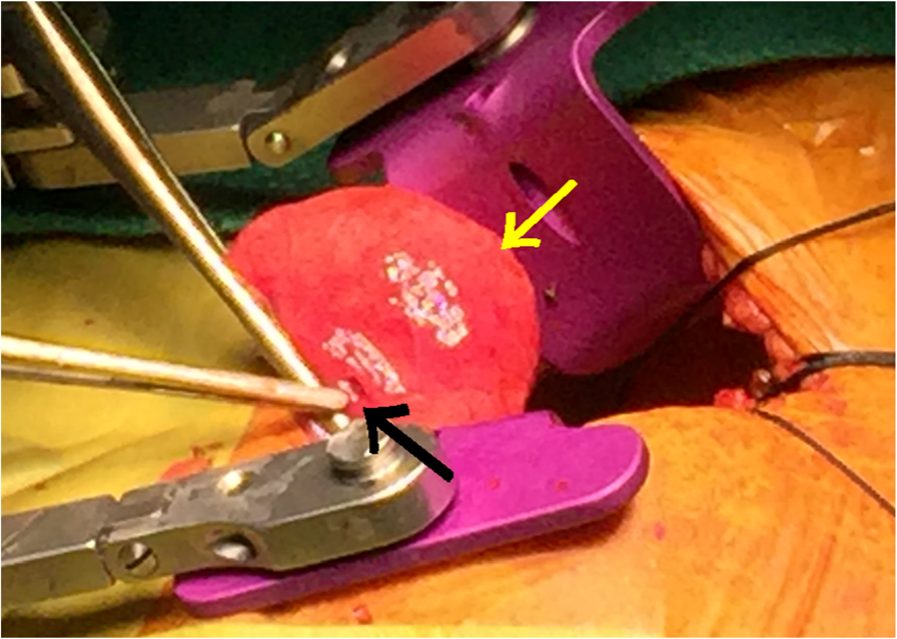
An intraoperative view of the left upper lobe. An operative image showing the perforation site (black arrow) in the left upper lingular segment, with swollen lung (yellow arrow) containing a pulmonary cyst

Thoracotomy was performed at the left anterior 5th intercostal space. Anatomical location of RV lead perforating the apex, with a neighboring swollen area, was consistent with the CT images that presented with the areas of perforation with pneumatocele (Fig. 3). To remove RV lead from the perforated RV apex, RV lead was detached from the generator, and U shape suture with Prolene™ 4-0 sutures (Ethicon Inc., Somerville, NJ, USA) was placed around the site of perforation. The suture was reinforced with Prolene™ 4-0 sutures as the lead was extracted from the cardiac wall. Subsequently, the swollen portion of the injured left upper lingular lung segment was resected to prevent worsening of pneumothorax or infection. A new RV lead placement was avoided because the patient demonstrated normal atrioventricular conduction. Pathology of the resected specimen confirmed an aseptic pneumatocele. The clinical course was uneventful. The patient returned to the outside hospital in a stable condition on postoperative day 9.

## Discussion

We report a rare case of RV perforation, pneumatocele and pneumothorax after a pacemaker lead placement, requiring partial lung resection.

RV perforation from pacemaker lead placement occurs rarely (0.3–3%) [1, 3–5]. In contrast to subacute perforation (24 h–1 month after implantation), acute perforation (within 24 h after implantation) with hemodynamic compromise warrants immediate attention. Surgery may be best chosen because of neighboring organ injury, or hemodynamic compromise due to acute cardiac tamponade in the setting of perforated lead removal [2, 6]. On the other hand, in stable conditions, simple direct traction can be considered under close echocardiographic monitoring and with a surgical backup [2, 3, 6]. Because the right heart is a low-pressure system, a perforation may be sealed by the lead itself and/or a combination of muscle contraction and fibrosis over the lead, with minimum sequelae [3, 4].

Strategies should depend on the dynamics of symptoms, pericardial effusion, hemodynamic status, and injured neighboring organs [2, 6].

When the patient is pacing-dependent, lead extraction should be followed by new lead placement in a different location, preferably in the RV outflow tract or the intraventricular septum. In the case of open-chest surgery, the implantation of epicardial leads may be considered [2].

RV lead replacement was avoided in our patient due to patent atrioventricular conduction, considering that the AAI pacing can achieve a clinical outcome similar to that of the DDD. Pneumothorax is a potential complication of vascular access during a pacemaker implantation (0.2–3.87%) [1, 5], frequently seen within the first 24 h after the implantation (1.3–3.87%) [5, 7]. On the other hand, pacemaker lead penetrating the myocardium and causing pneumothorax is rare and usually is found over 24 h after pacemaker implantation [3].

Generally, management of pneumothorax is guided by the amount of air and patient’s hemodynamic status [8]. A chest tube should be considered when the patient has respiratory distress, hemopneumothorax, or any pneumothorax larger than 20% of the hemithorax, irrespective of the symptoms [8]. Our patient presented with a pneumothorax that involved almost 10% of the pleural cavity.

Pathological analysis of the resected lung specimen revealed that the cystic space on CT imaging was a pneumatocele. Pneumatocele is an air-filled cystic cavity in the lungs, and frequently caused by severe pneumonia, blunt thoracic trauma, chronic obstructive pulmonary disease, or hydrocarbon ingestion with aspiration [9–12]. Although several mechanisms have been proposed for the development of pulmonary pneumatocele, the exact reasons are unknown [9–11]. On the other hand, pneumatoceles may occur when bronchial injury or inflammation creates a check-valve mechanism for air entry into the lung parenchyma [9, 11, 12]. In general, pneumatocele is a benign, self-limited condition that rarely requires surgical intervention [13]. However, life-threatening tension pneumatocele with rapid enlargement can result in rupture and pneumothorax. Secondary infection may require surgical interventions [10, 12]. There are no well-established or widely accepted treatment algorithms for pneumatocele [12, 13].

Pneumatocele due to RV pacemaker lead perforation has scarcely been reported, thus, standard of care remains to be determined. In the present case, surgery was chosen because of its potential for infection and pneumothorax, and surgery has better outcomes to avoid recurrent pneumothorax, compared with conservative treatment [14].

## Conclusions

Pneumothorax and pneumatocele associated with RV pacemaker lead perforation is extremely rare, therefore, the treatment is not well established. In our patient, a radical surgical intervention provided an excellent outcome.

## Data Availability

Not applicable.

## Abbreviations

RV: Right ventricular
CT: Computed tomography
